# EARLY REFEEDING AFTER COLORECTAL CANCER SURGERY REDUCES COMPLICATIONS AND LENGTH OF HOSPITAL STAY

**DOI:** 10.1590/0102-6720202400060e1854

**Published:** 2025-01-20

**Authors:** Eliani Frizon, José Eduardo de Aguilar-Nascimento, Júlio Cesar Zanini, Mariah Steinbach Roux, Bruna Caroline de Lima Schemberg, Pamela Luiza Tonello, Diana Borges Dock-Nascimento

**Affiliations:** 1 Universidade Federal da Fronteira Sul, Nutrition Course – Realeza (PR), Brazil; 2 Universidade Federal do Mato Grosso, Faculty of Medicine, Postgraduate in Health Sciences – Cuiabá (MT), Brazil; 3 Centro Universitário de Várzea Grande, Faculdade de Medicina – Várzea Grande (MT), Brazil; 4 Hospital do Câncer de Cascavel, Department of Digestive Surgery and Nutrition – Cascavel (PR), Brazil.

**Keywords:** Enhanced recovery after surgery, Colon, Fasting, Length of stay, Postoperative care, Recuperação pós-cirúrgica melhorada, Colon, Jejum, Tempo de internação, Cuidados pós-operatórios

## Abstract

**BACKGROUND:**

Multimodal protocols such as Acceleration of Total Postoperative Recovery and Enhanced Recovery After Surgery propose a set of pre- and post-operative care to accelerate the recovery of surgical patients. However, in clinical practice, simple care such as early refeeding and use of drains are often neglected by multidisciplinary teams.

**AIMS:**

Investigate whether early postoperative refeeding determines benefits in colorectal oncological surgery; whether the patients’ clinical conditions preoperatively and the use of a nasogastric tube and abdominal drain delay their recovery.

**METHODS:**

Retrospective cohort carried out at the Cascavel Uopeccan Cancer Hospital, including adult cancer patients (age ≥18 years), from the Unified Health System (SUS), who underwent colorectal surgeries from January 2018 to December 2021.

**RESULTS:**

275 patients were evaluated. Of these, 199 (75.4%) were refed early. Late refeeding (odds ratio — OR=2.1; p=0.024), the use of nasogastric tube (OR=2.72; p=0.038) and intra-abdominal drain (OR=1.95; p=0.054) increased the chance of infectious complication. Multivariate analysis showed that receiving a late postoperative diet is an independent risk factor for infectious complications. Late refeeding (p=0.006) after the operation and the placement of an intra-abdominal drain (p=0.007) are independent risk factors for remaining hospitalized for more than five days postoperatively.

**CONCLUSIONS:**

Refeeding early in the postoperative period reduces the risk of infectious complications. Using abdominal drains and refeeding late (>48h) for cancer patients undergoing colorectal surgery are risk factors for hospital stays longer than five days.

## RESUMO

**RACIONAL:**

Protocolos multimodais como o de Aceleração da Recuperação Total Pós-operatório e o Enhanced Recovery After Surgery propõem um conjunto de cuidados pré e pós-operatório para acelerar a recuperação dos pacientes cirúrgicos, contudo, na prática clínica cuidados simples como realimentação precoce e uso racional de drenos são muitas vezes negligenciados pelas equipes multiprofissionais.

**OBJETIVOS:**

investigar se a realimentação precoce, no pós-operatório, determina benefícios em cirurgia oncológica colorretal; se as condições clínicas do paciente no pré-operatório e o uso de sonda nasogástrica e dreno abdominal atrasam a recuperação do paciente.

**MÉTODOS:**

Coorte retrospectiva realizada no Hospital do Câncer de Cascavel (Uopeccan), incluindo pacientes adultos (idade ≥18 anos), oncológicos, do Sistema Único de Saúde (SUS), submetidos a cirurgias colorretais de janeiro de 2018 a dezembro de 2021.

**RESULTADOS:**

Foram avaliados 275 pacientes. Destes 199 (75,4%) foram realimentados precocemente. A realimentação tardia (OR=2,1; p=0,024), o uso de sonda nasogástrica (OR=2,72; p=0,038) e dreno intra-abdominal (OR=1,95; p=0,054) aumentaram a chance de complicação infecciosa. A análise multivariada mostrou que receber dieta tardia no pós-operatório é fator de risco independente para complicação infecciosa. A realimentação tardia (p=0,006) após a operação e a colocação de dreno intra-abdominal (p=0,007) são fatores de risco independentes para permanecer mais que cinco dias internados no pós-operatório.

**CONCLUSÕES:**

realimentar precocemente no pós-operatório diminui o risco de complicações infecciosas, assim como usar drenos abdominais e realimentar tardiamente (>48 h) pacientes oncológicos submetidos a cirurgias colorretais são fatores de risco para permanência hospitalar superior a cinco dias.

## INTRODUCTION

Multimodal protocols such as the Acceleration of Total Postoperative Recovery (*Aceleração da Recuperação Total Pós-Operatória* — ACERTO)^1,4–6^ and Enhanced Recovery After Surgery (ERAS)^21^ propose, among other recommendations, early oral refeeding (EORF) as one of the approaches to accelerate the recovery of surgical patients. These protocols also rationalize the use of abdominal drains and do not indicate the insertion of a nasogastric tube, as a preventive measure for complications^6,21^.

Studies show, especially in colorectal surgeries, that EORF is efficient in accelerating the resolution of adynamic ileus^7,34,35,44^, resuming solid food consumption^7,12,31^, decreasing postoperative intravenous hydration^31^, reducing postoperative complications^13,22,38,43^, length of hospital stay^7,22,23,40,43^ and hospital costs^22,27,44^. The safety of this recommendation is consistently supported in the literature by several studies and meta-analyses^12,31,35,45^. In addition, EORF improves patient satisfaction and well-being^35^ and immune function^22^ and does not increase anastomotic dehiscence^2,34,45^. On the other hand, the practice of prolonged fasting, common in digestive tract surgeries, increases metabolic stress, thirst, hunger, nitrogen losses^40^, intestinal permeability^16,37^ and worsens nutritional status^29,43^. In oncological surgery, where the patient has an increased nutritional risk, prolonged fasting is even more harmful^15^.

Given the current recommendations for EORF in colorectal surgeries^2,3,9,12,13,17,28^, the present study had the main objective of investigating whether EORF, in the postoperative period, determines benefits in colorectal oncological surgery. As a secondary objective, it was also investigated whether the patient's clinical conditions in the preoperative period and the use of a nasogastric tube and abdominal drain delay the recovery of patients undergoing colorectal surgery.

## METHODS

This was retrospective cohort study carried out at the Cancer Hospital of Cascavel (PR), from January 2018 to December 2021, including adult patients (age ≥ 18 years), oncology patients, from the Unified Health System. The study was approved by the Research Ethics Committee of the Universidade Federal da Fronteira Sul (number 4.861.48 of 2021).

The electronic medical records of oncology patients who underwent elective colorectal surgeries performed by laparotomy, of medium and large size, were included. The medical records of patients who underwent video-assisted surgery, those whose data did not meet the objectives of the study or were incomplete, and those under private care or insured were excluded, so that the sample included only patients from the Unified Health System.

The main outcome variable was the frequency of infectious complications in the postoperative period. Secondary outcome variables included length of hospital stay — LOS (days, categorized as ≤5 days), mortality, and unplanned readmission within 30 days postoperatively. Other variables collected were age, sex, muscle mass index (body mass index — BMI; kg/m^2^), nutritional risk by the Nutritional Risk Score-2002 (NRS-2002)^29^, physiological status (American Society of Anesthesiologists — ASA)^33^, type of operation, operation time (minutes), postoperative fasting time (hours), time to start early oral diet (up to 48 hours), type of oral diet prescribed in the postoperative period. Patients aged ≥60 years were considered elderly.

The postoperative infectious complications considered were the presence of pneumonia, surgical site and urinary tract infection, anastomotic or wall dehiscence, abscess, enterocutaneous fistulas, sepsis and peritonitis^8^.

For statistical purposes, the presence of infectious complications, postoperative mortality, unplanned readmission within 30 days after surgery and LOS were statistically associated with early (≤48 h) or late (>48 hours) refeeding; whether the patient was elderly or not, whether the ASA score was below or above 2, whether or not a nasogastric tube was used and finally whether or not an intra-abdominal drain was placed.

The Kolmogorov-Smirnov test was used to determine the normality of continuous data. If normally distributed, they were summarized as mean and standard deviation (M±SD), and, if asymmetrically distributed, as median (M) and interquartile range (IQR). The Student's t-test (M±SD) or Mann-Whitney (M; IQR) was used to compare the studied variables with the LOS in days. The chi-square test or Fisher's exact test was used to determine the association between the studied variables. The association was presented as a percentage, odds ratio (OR), and the respective 95% confidence interval (CI). The multivariate logistic regression model was constructed to explore whether or not the risk factors were independent predictors for infectious complications and postoperative hospital stay greater than five days. The variables that presented an association with p-value <0.20 in the univariate comparison were entered into the multivariate logistic regression model (stepwise model). For multivariate analysis, postoperative hospital stay was categorized as ≥5 days. A significance limit of 5% (p=0.05) was established. The Statistical Package for the Social Sciences 20.0 (SPSS Statistics; IBM, Armonk, NY, USA) was used for statistical analyses.

## RESULTS

**Figure 1:**
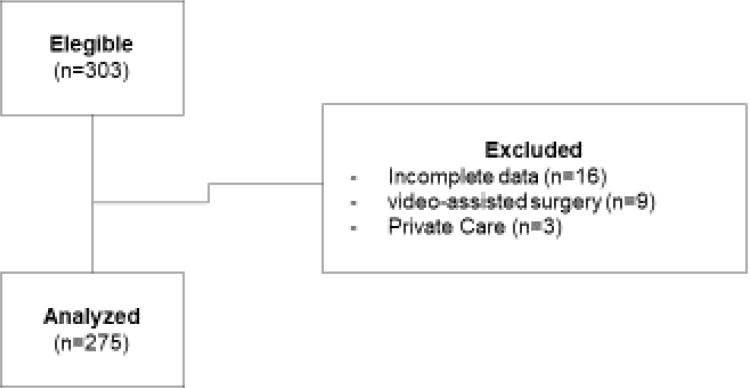
Flowchart of eligible patients who took part in the study.

**Figure 2:**
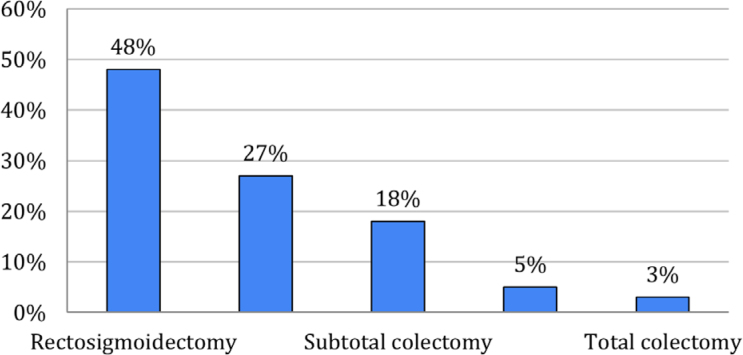
Type of colorectal oncological surgery performed among the patients studied.

**Figure 3:**
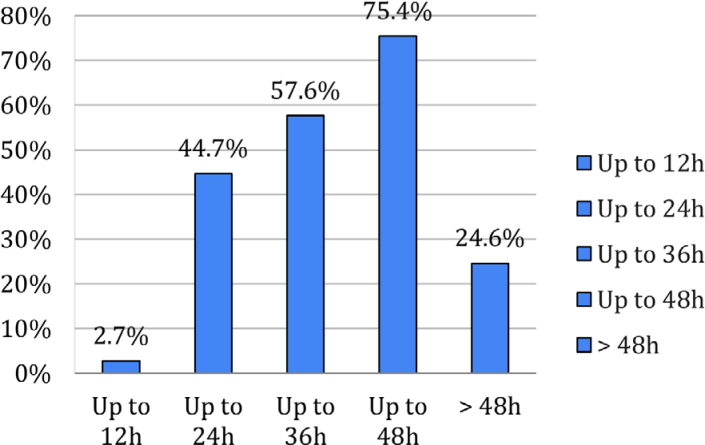
Early initiation of oral diet (hours) in the postoperative period of cancer patients undergoing colorectal surgery.

All patients underwent open colorectal surgery by the same team of surgeons and anesthesiologists according to the hospital's surgical schedule. Initially, 303 medical records were eligible; however, 28 were excluded and, in the end, 275 met the eligibility criteria and were analyzed (Figure 1). The mean age of patients was 60 (±13.2) years, and 144 (52.9%) were male. The data characterizing the patients studied are described in Table 1. As shown in Figure 2, rectosigmoidectomy was the most frequently performed surgery (48%; n=132). In the first 48 hours postoperatively, 75.4% (n=199) of patients received early oral nutrition (Figure 3).

**Table 1:** Characteristics of the studied oncology patients undergoing colorectal surgery. Results presented as mean and standard deviation, median and interquartile range or as absolute numbers and frequency.

Variables		Values
Age (years; M±SD)		60±13,2
	Elderly (≥ 60 years)	148 (53.8)
Gender (n;%)		
	Feminine	128 (47.1)
	Masculine	144 (52.9)
BMI (kg/m^2^ — M; IQR)		25.5 (22.4–28.5)
Nutritional risk (n;%)		56 (25.9%)
ASA I e II		236 (91.8)
Operation time (minutes — M; IQR)		4 (2.5–4.5)
Postoperative fasting time (h — M; IQR)		25.2 (20.2–47.2)
Postoperative liquid/restricted liquid prescribed oral diet (n;%)		229 (83.3)
Use of NGT (n;%)		20 (7.3)
Placement of intra-abdominal drain (n;%)		168 (61.1)
Infectious complications (n;%)		49 (17.9)
Total postoperative hospital stay (days — M;IQR)		4 (3-6)
Length of hospital stay ≤5 days (n;%)		194 (70.8)
Readmission in the 30 days post-operatively (n;%)		51 (18.5)
Mortality (n;%)		13 (4.72)
Age (years; M±DP)		60±13.2

n: numbers; %: frequency; M±SD: mean and standard deviation; BMI: body mass index; M: median; IQR: interquartile range; ASA: American Society of Anesthesiologists; NGT: nasogastric tube.

Late initiation of refeeding (5 [5–7] vs. 4 [3–5] days; p<0.001) and placement of intra-abdominal drain (5 [4–7] vs. 3 [2–4] days; p=0.001) resulted in one to two days more postoperative hospital stay, respectively. The same was not observed for age ≥60 years (p=0.213), ASA >2 (p=0.226) and use of nasogastric tube (p=0.287) (Tables 2 and 3).

**Table 2:** Association of delayed refeeding, age ≥60 years (elderly), American Society of Anesthesiologists score with postoperative outcomes in cancer patients undergoing colorectal surgery

Outcomes	Late refeeding				Elderly				ASA>2			
	Yes (%)	No (%)	OR 95%CI	p-value	Yes (%)	No (%)	OR 95%CI	p-value	Yes (%)	No (%)	OR 95%CI	p-value
Infectious Complications	26.2	14.1	2.10 (1.10–4.30)	0.024	23.0	11.9	2.2 (11.14–4.3)	0.017	33.3	15.7	2.7 (1.01–7.08)	0.041
Readmission	18.5	18.1	1.00 (0.49–2.11)	0.946	18.9	18.1	1.0 (0.57–1.94)	0.863	9.5	19.1	0.45 (0.10–2.00)	0.222
Mortality	3.1	4.0	0.76 (0.16–3.70)	0.536	6.8	2.4	3.0 (0.81–11.1)	0.075	4.8	4.2	1.13 (0.14–9.30)	0.616
Post-op LOS (days – M;IQR)	5 (5–7)	4 (3–5)	-	<0.001	5(3-7)	4(3-5)	-	0.213	5(3-5)	4(3-6)	-	0.226

ASA: American Society of Anesthesiologists; OR: odds ratio CI: confidence interval; Post-op LOS: Postoperative length of stay. Chi-square or Fisher's exact test.

**Table 3:** Association of the use of nasogastric tube and intra-abdominal drain with postoperative outcomes in cancer patients undergoing colorectal surgery.

Outcomes	Use of NGT				Placement of intra-abdominal drain			
	Yes (%)	No (%)	OR 95%CI	p-value	Yes (%)	No (%)	OR 95%CI	p-value
Infectious complications	35	16.5	2.72 (1.02–7.22)	0.038	21.4	12.6	1.95 (0.98–3.90)	0.054
Readmission	20	18.4	1.11 (0.35–3.46)	0.528	20.8	15.0	1.50 (0.78–2.86)	0.221
Mortality	15	3.9	4.32 (1.10–17.20)	0.059	4.2	5.6	0.73 (0.24–2.24)	0.538
Post-op LOS (days – M;IQR)	5 (3–14)	4.0 (3–6)	-	0.287	5 (4–7)	3 (2–4)	-	0.001

NGT: nasogastric tube; OR: odds ratio CI: confidence interval; Post-op LOS: Postoperative length of stay. Chi-square or Fisher's exact test.

Univariate analysis showed that late refeeding (>48 h) increased the chance of infectious complications by two times (OR=2.1; p=0.024). Also, being elderly (OR=2.2; p=0.017) or having an ASA score >2 (OR=2.7; CI, p=0.041) increased the chance of the patient presenting an infectious complication after the operation (Table 2). The use of nasogastric tube (p=0.038) and intra-abdominal drain (0.054) were associated with a higher chance of postoperative infectious complications than those who did not require this prescription (Table 3). As shown in Table 3, the use of nasogastric tube was associated with increased postoperative mortality (p=0.059).

Multivariate logistic regression analysis showed that age ≥60 years (p=0.029), receiving a late diet (>48 h) in the postoperative period (p=0.014) and having an ASA score >2 (p=0.040) were independent risk factors for infectious complications in the postoperative period (Table 4). Multivariate analysis also showed that late refeeding (>48 h) (p=0.006) in the postoperative period and placement of an intra-abdominal drain (p=0.007) were independent risk factors for staying in the hospital for more than five days in the postoperative period (Table 5).

**Table 4:** Multivariate logistic regression analysis for the association of infectious complications with the variables studied among cancer patients undergoing colorectal surgery.

Variable	OR	95%CI	p-value
Age ≥60 years (elderly)	2.33	1.1–5.0	0.029
Late refeeding >48h	2.64	1.22–5.71	0.014
Use of NGT	2.65	0.74–9.41	0.132
ASA Score > 2	3.05	1.05–8.86	0.040
Intra-abdominal drain	1.66	0.74–3.76	0.220

NGT: nasogastric tube; ASA: American Society of Anesthesiologists; OR: odds ratio; CI: confidence interval.

**Table 5:** Multivariate logistic regression analysis for the association of hospital stay longer than five days with the variables studied among cancer patients undergoing colorectal surgery.

Variable	OR	95%CI	p-value
Late refeeding >48 h	2.35	1.28–4.32	0.006
Intra-abdominal drain	2.39	1.27–4.50	0.007

OR: odds ratio; CI: confidence interval.

## DISCUSSION

The data from the present study show that EORF was initiated postoperatively for more than 75% of patients. Patients with EORF benefited significantly from a lower chance of postoperative infectious complications, in addition to remaining hospitalized for one day less than those with a late diet initiated over 48 hours after colorectal surgery^41^. On the other hand, late initiation of the oral route, after 48 hours, resulted in harm to patients, being an independent risk factor for infectious complications and for remaining significantly more than five days hospitalized in the postoperative period. This result corroborates other studies^7,10,22,23,31,34^ that showed a longer hospital stay for patients who were re-fed late and with the results of Assis et al.^13^, who state that the longer the postoperative fasting time, the greater the risk of infections. Recently, a meta-analysis^22^ involving 2,100 patients after upper gastrointestinal tract surgeries showed a lower risk of pneumonia, better immune function, and shorter hospital stay for those fed up to 24 hours after surgery. Other studies^17,22,28,38^ also showed a lower risk of general complications in patients who were fed early after various types of surgeries.

This "apparently" simple act of introducing food and nutrients into the intestinal lumen modulates the complex physiological processes caused by surgical trauma^2,25^ with a lower inflammatory response^24^ and, consequently, lower proteolysis and nitrogen loss^40^. In addition, it promotes the integrity of the intestinal mucosa^16,26,37^ and the balance of the microbiota^16^. In this sense, the ACERTO protocol recommends the early initiation of a liquid oral diet for colorectal surgeries^3^. This diet rich in fluids, electrolytes and nutrients such as carbohydrates and especially proteins can significantly contribute to the reduction of worse outcomes and 30% of postoperative mortality^32^.

In this context, a recent study by Franco et al.^18^, conducted with 154 patients, showed that an oral diet initiated in the anesthesia recovery room, called "ultra early", brought important benefits to patients. This clarified liquid diet, rich in proteins, in addition to showing good adherence for 93.5% of patients, significantly reduced the volume of intravenous fluid, resulting in a lower need for hydration and less time in the ward for the evolution of the hospital oral diet.

Increasingly, scientific evidence points to the importance of maintaining nutrients in the intestinal lumen in order to minimize the effects of surgical trauma. A groundbreaking study comparing fasting patients with those who did not have their diet interrupted during surgery and received 10 mL/h of nutrition via nasogastric tube during intraoperative head and neck cancer with free flap reconstruction observed no differences in total or partial flap failure, but significantly lower rates of wound dehiscence or edge necrosis were observed^24^. In addition, this study showed that intraoperative nutrition modulates marked inflammation with lower concentrations of interleukins IL6 and IL8 after surgery^24^.

Infectious complications observed in the present study were also associated with age ≥60 years, ASA score >2, use of nasogastric tube and insertion of drains. Regarding advanced age, in a cohort^36^ of 3,849 adult and elderly Swedish patients undergoing colorectal resection for cancer, no differences were observed in relation to complications, reoperations or postoperative readmissions. However, the study showed more than twice the chance of death within 90 days among the elderly (>70 years).

In this scenario, what may explain the difference between the Swedish study and ours is the better quality of life of that population. Aging in that country does not compromise the physical and nutritional status of individuals as much as it does here in Brazil and in other developing or underdeveloped countries.

In addition, elderly individuals are affected by a phenomenon described as immunosenescence, which is characterized by innate or adaptive immune dysfunction and increased susceptibility to infections, among other physiological changes^30^. When these individuals need to undergo surgery, they become vulnerable to infections, as shown in the present study. In this sense, special attention should be given to the diet of elderly individuals, as it is a modifiable factor that positively affects recovery. Early refeeding after surgery should be part of the nutritional approach for these patients^9^.

Furthermore, having an ASA score greater than 2 was associated with greater postoperative complications. Among the possible classifications of the ASA score^33^, a score greater than 2 indicates the presence of poorly controlled underlying diseases that in themselves increase the risk to patients^19^. Thus, it is expected that they will have more complications and perioperative mortality.

Late refeeding (>48 hours) has been shown to be an independent risk factor for hospital stays longer than five days. This result was found in several other studies^7,10,22,23,31,34,42^ that showed longer hospital stays for patients who were refed late. In 1999, Kehlet et al.^28^ showed that early refeeding, within the first 48 hours after surgery, not only contributes to a shorter hospital stay (an average of two days), but also to an earlier return to bowel movement, with lower fatigue and pain scores during the first days after surgery.

What may explain a shorter hospital stay with early release of the diet in the postoperative period is the return of intestinal peristalsis. Although in many services, traditionally, the release of the oral diet in the postoperative period is conditioned only on the presence of signs of resolution of the ileus with elimination of flatus and feces, there is no evidence to support this conduct^41^.

In light of the evidence, there is no doubt that EORF is a stimulus for the return of intestinal peristalsis, which stimulates hunger and consequently the release of flatus and feces^7,34,35,44^ more quickly in the postoperative period, especially in colon and rectal surgeries, in which the ileus time tends to be longer^1,2,12^. Ashcroft et al.^7^ showed, through a meta-analysis, that EORF is the most effective therapy for reducing postoperative ileus in patients undergoing colorectal surgery. Some authors^12,18,31^ observed that EORF accelerates the return of solid food intake, which in turn contributes to a more adequate supply of nutrients in the postoperative period.

Furthermore, the use of intra-abdominal drains was an independent risk factor for increased hospitalization, corroborating other studies^14,20^. On the other hand, the positive and negative implications on morbidity and mortality remain a controversial subject^11,14^. In a recent review, the authors^39^ point to the need for studies that can refine patient selection criteria and determine the timing and duration of drain use.

Although the present study significantly demonstrated the clinical benefits of earlier oral diet release in the postoperative period, it has limitations. First, it is a retrospective investigation, the nutritional status of the patients in the preoperative period was not observed, and second, the quality of the oral diet released in the postoperative period was also not recorded. The composition of the diet in EORF can impact outcomes, since a greater amount of proteins determines better results. However, we can state that, even with the limitations of the study, the present investigation showed the benefits of early release of the oral diet in the postoperative period in colorectal cancer surgeries.

## CONCLUSIONS

It is concluded that EORF is beneficial in reducing the rate of infectious complications and the length of postoperative hospital stay. Furthermore, the use of intra-abdominal drain was an independent risk factor for increasing the length of postoperative hospital stay in oncology patients undergoing colorectal surgery.

Central Message This study showed that early oral feeding (≤48 h) reduced infectious complications and length of hospital stay in cancer patients undergoing colorectal surgery. On the other hand, oral feeding after 48 hours and use of intra-abdominal drains were independent risk factors for hospital stay ≥5 days. These results are in agreement with current evidence based on multimodal protocols to accelerate post-surgical recovery, such as the Acceleration of Total Postoperative Recovery (*Aceleração da Recuperação Total Pós-Operatória* — ACERTO) Project. The ACERTO Project recommends starting an early oral diet as one of the main approaches to accelerate the recovery of surgical patients in the postoperative period.

Perspectives Early refeeding contributes to the accelerated recovery of surgical patients with a lower risk of complications, which consequently contributes to a reduction in the length of hospital stay. This approach should be implemented in all units caring for surgical patients undergoing colorectal surgery. In this concept of early feeding, the approach known as "ultra-early oral feeding" is increasingly being used, starting in the post-anesthesia recovery room. Neglecting this approach not only delays the recovery of surgical patients with more complications, but also increases the length of hospital stay and hospital costs.
